# Refining the Results of a Classical SELEX Experiment by Expanding the Sequence Data Set of an Aptamer Pool Selected for Protein A

**DOI:** 10.3390/ijms19020642

**Published:** 2018-02-24

**Authors:** Regina Stoltenburg, Beate Strehlitz

**Affiliations:** 1 UFZ—Helmholtz Centre for Environmental Research, Department of Soil Ecology, 06120 Halle, Germany; 2 UFZ—Helmholtz Centre for Environmental Research, Department of Environmental and Biotechnology Centre, 04318 Leipzig, Germany; beate.strehlitz@ufz.de

**Keywords:** aptamer, Protein A, *Staphylococcus aureus*, SELEX, NGS, sequence analysis, binding affinity, SPR

## Abstract

New, as yet undiscovered aptamers for Protein A were identified by applying next generation sequencing (NGS) to a previously selected aptamer pool. This pool was obtained in a classical SELEX (Systematic Evolution of Ligands by EXponential enrichment) experiment using the FluMag-SELEX procedure followed by cloning and Sanger sequencing. PA#2/8 was identified as the only Protein A-binding aptamer from the Sanger sequence pool, and was shown to be able to bind intact cells of *Staphylococcus aureus*. In this study, we show the extension of the SELEX results by re-sequencing of the same aptamer pool using a medium throughput NGS approach and data analysis. Both data pools were compared. They confirm the selection of a highly complex and heterogeneous oligonucleotide pool and show consistently a high content of orphans as well as a similar relative frequency of certain sequence groups. But in contrast to the Sanger data pool, the NGS pool was clearly dominated by one sequence group containing the known Protein A-binding aptamer PA#2/8 as the most frequent sequence in this group. In addition, we found two new sequence groups in the NGS pool represented by PA-C10 and PA-C8, respectively, which also have high specificity for Protein A. Comparative affinity studies reveal differences between the aptamers and confirm that PA#2/8 remains the most potent sequence within the selected aptamer pool reaching affinities in the low nanomolar range of *K_D_* = 20 ± 1 nM.

## 1. Introduction

It is now more than 25 years since the advent of a new kind of affinity molecules called aptamers [1,2]. During this time, they have received a growing interest in diverse scientific fields. Aptamers are artificial short single-stranded DNA or RNA molecules that are selected for recognition and binding of a specific target. To date, aptamers have been described for a wide variety of target molecules ranging from small molecules to proteins and composites of targets. The ability to fold into a unique three-dimensional structure is necessary for their molecular interactions with the target and for forming a stable aptamer-target complex. These features make aptamers functionally comparable to the widely used antibodies, but they also offer several notable advantages like high availability by chemical synthesis, none or low batch-to-batch variation, versatile chemical modification, high storage stability, regenerability, none or low immunogenicity, small size, flexible structure, and high affinity and specificity to their target, that can compete with that of antibodies [3]. Aptamers are currently in great demand in many fields of application from basic to medical and pharmaceutical research, or in the different areas of analytics. Their high potential as therapeutic or diagnostic agents, delivery agents, molecular imaging tools, as capture or reporter molecules in analytical systems, and as recognition elements in biosensors has been demonstrated [3,4,5,6,7,8]. 

Aptamers are generated by an in vitro method termed SELEX. This evolutionary approach leads to the enrichment of specific target-binding aptamers from a highly diverse oligonucleotide library with up to 10^15^ different molecules during iterative selection cycles [9,10,11]. The SELEX technology has become a powerful standard method for the selection of high-affinity aptamers, and has been extensively modified and optimized over the years. There are many variations especially with respect to the composition of the SELEX library, the adaption of process conditions for directional selection of aptamers with desired features, and the efficient separation of unbound from target-bound oligonucleotides and their recovery as the most crucial steps of a SELEX process. Significant progresses were achieved in optimizing specific technical protocols or introducing new methods and automatic processes. There are an ongoing number of comprehensive reviews addressing the current status of the SELEX technology and highlighting the recent progress but also giving main obstacles and limitations of the process [9,12,13,14,15,16,17].

A classical SELEX experiment involves multiple rounds of selection (usually 6–20) to get at the end an oligonucleotide pool enriched with target-specific aptamers. After cloning and Sanger sequencing of this aptamer pool, individual aptamers are chosen and comprehensively characterized and optimized during the post-SELEX phase. The whole process is very laborious and time-consuming. Therefore, most of the modifications aim at the improvement and acceleration of the aptamer selection process [12,13]. In this regard, NGS technologies have contributed substantially in the last years [15,18,19,20,21]. NGS has the potential to completely replace cloning and Sanger sequencing. It can be applied in a low to high throughput format. Clustering and alignment methods allow the analysis of the frequency distribution of sequences in the data set and the formation of sequence families. Moreover, in combination with more complex bioinformatics tools for analyzing millions of sequences, NGS enables a deep insight into the dynamics of a selection process by applying it to each selection round. It allows monitoring of the enrichment of certain sequences or subsequences by tracking their frequency over the entire selection process. The expanded sequence space by NGS also allows the identification of rare aptameric sequences. The inclusion of structure prediction tools gives information about the enrichment of essential structural motifs. Finally, the impact of different selection conditions on the outcome of a SELEX experiment can be studied more easily.

NGS can help to reduce the time required for an aptamer selection process by focusing on only a few selection rounds. But this is only one part of the whole aptamer development. There is mostly little awareness of the post-SELEX phase among the SELEX beginners or end users of aptamers [11,22]. The selection process generally results in a large number of potent aptameric sequences, which need to be evaluated individually for their binding to the target. The most promising sequences are then chosen as candidate aptamers for an extensive characterization in terms of their target-binding affinities and specificities, their structural features including sequence optimizations and modifications, and their functionalities under different binding conditions. This is a very costly and time-consuming process, but absolutely necessary for the identification of the best and most suitable aptamer and hence for increasing the acceptance of aptamers and bringing them into real applications.

*Staphylococcus aureus* is a ubiquitous human pathogenic bacterium causing a range of diseases from minor skin infections to systemic and life-threatening diseases. It is known as one of the most common pathogens of nosocomial infections worldwide [23]. *S. aureus* is an extraordinarily adaptable bacterial species. This enables the bacteria to rapidly evolve new defense strategies to antibacterial drugs like antibiotics. As result, many of these drugs have become almost completely ineffective. Such antibiotic-resistant strains, called MRSA (methicillin-resistant *S. aureus*), are not only a risk of the hospital-associated infections, but also cause increasingly community-associated infections, and therefore represent a major public health problem [24,25]. Moreover, *S. aureus* is also recognized as one of the most common causes of food-borne diseases worldwide, because of its ability to produce enterotoxins [26]. Therefore, prevention and control of infection with *S. aureus* is a major health care focus. Standard detection methods are based on culturing the bacteria using selective media followed by biochemical identification methods. These are well-established methods, but they are time consuming and laborious. To overcome some limitations of the conventional methods, more rapid methods have been developed with increase in sensitivity and specificity, which can be categorized into immunological methods and molecular biology methods. Furthermore, biosensor technology in combination with the development of new biorecognition elements like aptamers has a great potential as a rapid, sensitive, cost-effective, and easy-operating detection method [27,28,29].

We have previously described the selection of DNA aptamers for the specific binding to Protein A as a cell surface component of *Staphylococcus aureus* [30]. We found one promising aptamer that was extensively characterized and evaluated for its ability to recognize and bind to Protein A in a whole cell context of the bacterial pathogen [31,32]. In this work, we applied NGS to the same selected aptamer pool to compare the outcome with that of Sanger sequencing and perhaps to expand our portfolio of Protein A-binding aptamers. This would give us more flexibility for our objective, the development of an aptamer-based assay for the detection of *S. aureus*.

## 2. Results and Discussion

### 2.1. Broadening the Sequence Data Set of the Aptamer Pool Selected for Protein A

**Figure 1:**
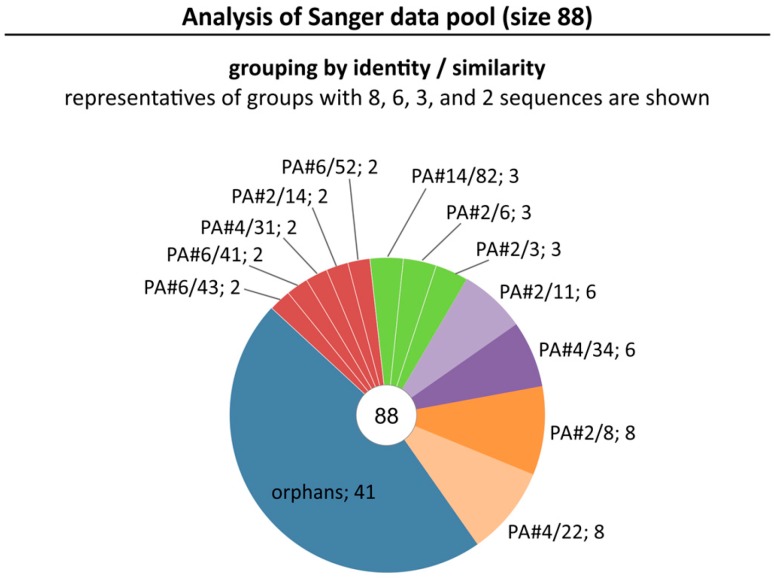
Selected pool of Protein A-binding aptamers after cloning and sequencing. Results of analysis of the Sanger data pool containing 88 sequences are shown. Sequences were grouped with respect to their identity and similarity. The representative of each group and the group size are indicated. Orphans represent sequences with no homology to any other sequence in this pool.

Previously, we described the process of DNA aptamer selection for Protein A known as a structural part of the cell wall of the bacterial pathogen *S. aureus* [30]. The selected aptamer pool after 11 rounds of the FluMag-SELEX procedure was further processed by the classical way of cloning and Sanger sequencing of individual aptamer clones. A data set of 88 sequences (Sanger data pool) was obtained (Figure 1). Identical and homologous sequences were grouped together resulting in 12 sequence groups. Each group consists of 2–8 sequences (total 47 sequences, 53.4%). In addition, 41 orphans (46.6%) were found which have no homology to one of the identified groups (Figure 1). Sequences from all groups were tested for target binding. Only one group with aptamer PA#2/8 as representative (8 group members, 9.1%) could be confirmed for binding to Protein A. These are rather unusual outcomes of a SELEX process indicating the selection of a very heterogeneous oligonucleotide pool missing a dominating sequence family.

**Figure 2:**
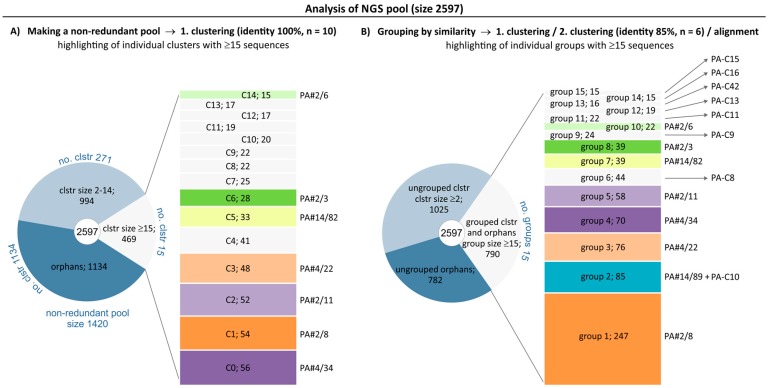
Selected pool of Protein A-binding aptamers after direct sequencing. Results of data analysis of the NGS pool containing 2597 sequences are shown. (**A**) First clustering of identical sequences resulted in a non-redundant pool of 1420 sequences (see also Figure S2). Clusters with a size of ≥15 sequences are highlighted (C0–C14) with the number of sequences included. Identified sequences from the Sanger data pool are indicated; (**B**) Second clustering (85% identity) of the non-redundant pool and a final alignment resulted in groups of sequences from the NGS pool with respect to their identity and similarity. Groups with a size of ≥15 sequences are highlighted (group 1–15) with the number of sequences included, which also determine the rank position of the group. The representative of each group is indicated and corresponds to the most frequent sequence in the group. It is named according to a known sequence from the Sanger data pool if identified or according to the cluster name from the non-redundant pool in case of new sequences.

Therefore, we additionally employed next generation sequencing technology for direct sequencing of the same aptamer pool to get a larger sequence data set. Bioinformatics tools were used to obtain information on the number and frequency of individual sequences, their grouping based on sequence similarities, group size and complexity. After pre-processing of the raw data set, there were 2597 sequences (NGS pool) to be analyzed by a two-step clustering and alignment method (Figure 2A,B and Figure S1). All identical sequences were clustered first resulting in a reduced non-redundant pool of 1420 sequences. This pool included 286 clusters with a size of ≥2 sequences (total 1463 sequences, 56.3%) and 1134 orphans (43.7%). All clusters with a size of ≥15 sequences (total 15 clusters C0–C14) were highlighted in Figure 2A. Seven representatives of sequence groups from the Sanger data pool (Figure 1) could be identified among these 15 clusters of the non-redundant NGS pool and are given on the right of the cluster boxes in Figure 2A. Moreover, the four largest groups and clusters of both data pools are represented by the same aptameric sequences PA#2/8 (C1), PA#4/22 (C3), PA#4/34 (C0) and PA#2/11 (C2). The remaining 8 of the 15 clusters were recognized as new aptameric sequences, because they were not present in the Sanger data pool. In addition to the 7 Sanger aptamer groups, all of the double sequences and also 22 orphans of the Sanger data pool could be identified in the non-redundant NGS pool (Figure S2). Only 19 orphans were not present in the NGS pool. These analysis results conform to that of the Sanger data pool and confirm the selection of a highly complex oligonucleotide pool. The proportion of orphans and the number of small clusters with a size of 2–5 sequences is very high. The relative frequency distribution of certain sequences in the NGS pool is comparable to that found for the Sanger data pool. There is no specific sequence with outstanding frequency as well.

**Figure 3:**
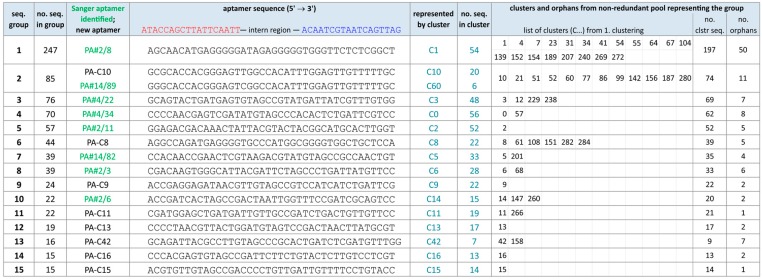
Identified sequence groups (size ≥ 15 seq.) from data analysis of the NGS pool by the two-step clustering and alignment method. All clusters coming from the non-redundant pool and the total number of orphans representing each group are listed.

A second clustering of the non-redundant NGS pool based on 85% sequence identity was performed followed by a final alignment. The combination of all results from clustering and alignment aimed at forming groups of homologous sequences from the whole NGS pool. The focus was on groups with at least 15 sequences, which were highlighted in Figure 2B. There are 15 groups (see also Figure 3) matching this condition and contain 790 sequences in total. 1025 another sequences belong to clusters but were not aligned yet for grouping, and 782 sequences (30.1%) remain orphans at this stage of data analysis.

The frequency distribution of sequences from the first clustering is reflected in the ranking of the identified sequence groups, whereby some significant changes could be observed. The four most abundant sequences in the NGS pool also represent the largest groups, group 1 (PA#2/8) and group 3–5 (PA#4/22, PA#4/34, PA#2/11). An exception forms group 2 with 85 sequences (3.3%), which is represented by a new aptameric sequence PA-C10. All new sequences that are not present in the Sanger data pool were named according to their cluster number (C…) from the first clustering. This means in case of PA-C10, it comes from cluster C10 with 20 identical sequences. Group 2 also contains a related sequence, which is already known as orphan PA#14/89 from the Sanger data pool and now exists six times in the NGS pool (Figure 3). By far the largest group with 247 sequences (9.6%) is group 1 represented by PA#2/8. This sequence is the only aptamer from the Sanger data pool able to bind Protein A. Representatives (PA#14/82, PA#2/3 and PA#2/6) of 3 further groups are known as multiple sequences from the Sanger data pool. Seven groups completely consist of new aptameric sequences. Therefore the NGS pool is clearly dominated by a sequence group in contrast to the Sanger pool.

### 2.2. Group Complexities and Consensus Sequences

Group 1 shows the highest sequence variability. The 247 sequences contained are represented by 20 differently sized clusters and 50 orphans (Figure 3). The most frequent sequence PA#2/8 (cluster C1) in this group was already analyzed in our previous work concerning its structural features and binding ability to Protein A [30,31]. It is characterized by four G-rich regions (GGGGG-D_5_-GGGGGTGGG-Y_7_-GG) and thus able to form a G-quadruplex structure, which we have demonstrated by circular dichroism spectroscopy [31]. Differences between the sequences of group 1 often concern these G-rich regions, especially the number of guanine residues (Figure S3). The size of each region varies, but is dominated by two (region 1 with G_5–6_) or only one variant (region 2–3 with G_5_TG_3_ and region 4 with G_2_) (Figure S4). Group 1 contains two further clusters C4 and C7 that also represent large clusters with ≥15 sequences. Cluster C4 (41 sequences) has the same G-profile as PA#2/8 (cluster C1), but differs at one nucleotide position in the sequence between G-rich region 1 and 2. In contrast, cluster C7 (25 sequences) contains 6 guanine residues in region 1 as only difference to PA#2/8. The high variability of this group may be caused by sequencing artefacts. The Roche 454 GS FLX system is known for its relatively high error rate in terms of homopolymers including insertions as the most common type of error [33,34,35]. Especially long stretches with more than three identical bases are affected, which is the case for at least two of the G-rich regions found with 5–6 guanines each.

Besides group 1, group 2 and 6 also contain several stretches of guanines, but in a completely different pattern (Figure 3 and Figure S5). The sequence variability in these groups is much lower than in group 1, but higher than in the other groups. Two consensus regions between PA-C8 (cluster C8) from group 6 and PA#2/8 from group 1 that overlap three G-stretches in both sequences were identified by sequence alignments (Figure S6). In contrast, no consensus regions were found between group 2 (PA-C10, cluster C10; PA#14/89, cluster C60) and PA#2/8 or PA-C8.

**Figure 4:**
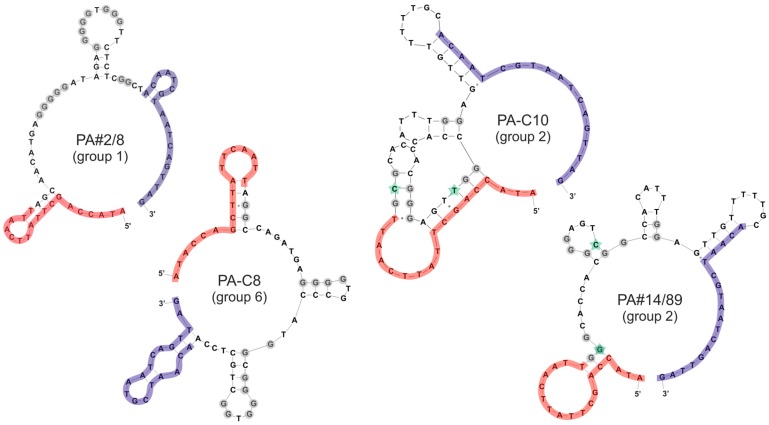
Potential secondary structures of sequences from the three G-rich sequence groups are given. The primer binding sites (18 nt each) at the 5′- and 3′-end are highlighted in red and blue, respectively. The G-stretches in the intern sequence regions are highlighted in grey. Two nucleotide positions that are different between PA-C10 and PA#14/89 are marked with a green star.

Figure 4 shows the predicted 2D-structures of the full-length sequences representing the three G-rich sequence groups. The close relationship between PA#2/8 and PA-C8 is affirmed by a common structural element at their 5′-ends forming a stem-loop. This element includes the 5′-primer binding region and was found to be essential for the target binding of PA#2/8 [30].

The other 12 groups are distinguished by their diversity without significant consensus regions (Table S1).

### 2.3. Functional Screening of Identified Aptamer Groups

**Figure 5:**
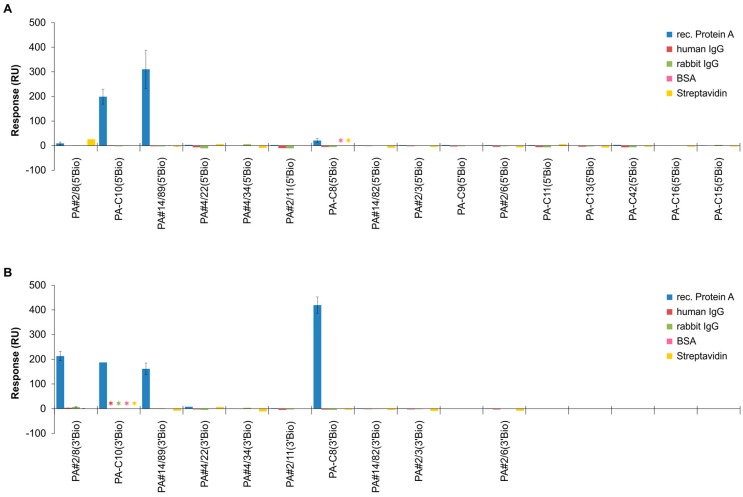
Representative sequences of the 15 identified groups from the NGS pool were screened for their individual binding abilities to Protein A. Comparative SPR-based interaction analyses were performed with the Biacore X100 instrument. Biotinylated aptamers were immobilized via the 5′-end (**A**) or 3′-end (**B**) on the streptavidin-modified sensor surface and 1000 nM Protein A was injected for binding. The sensor responses from the end of the binding phases (after 300 s) are shown. In addition, cross-specificities to other proteins were analyzed. Asterisks indicate if certain interactions have not been investigated.

The representatives of the identified 15 largest groups of homologous sequences from the NGS pool were screened for their individual binding abilities to Protein A. The SPR-based Biacore X100 instrument was used for these comparative interaction analyses where the 5′- or 3′-biotinylated aptamers were immobilized on a streptavidin-modified sensor surface. The results of interactions with Protein A are shown in Figure 5 revealing new functional aptamers in addition to the previously published ones. Besides group 1 with the already known aptamer PA#2/8, two further groups are able to bind Protein A: group 2 with PA-C10/PA#14/89 and group 6 with PA-C8. PA#14/89 is also known from the Sanger data pool but was not identified as binding sequence at that time. The other 12 groups do not show any interaction with Protein A. This indicates a high proportion of sequences in the selected aptamer pool not able to bind to the target, thus confirming the screening results of the Sanger data pool as described in Stoltenburg et al. [30]. Other proteins like streptavidin, bovine serum albumin (BSA) or immunoglobulins (human IgG, rabbit IgG) were applied to investigate alternative specificities of the selected sequences. But none of the 15 groups were able to bind to these proteins. This means, no cross reactivity for the Protein A-targeting aptamers from group 1, 2 and 6 were found. On the other hand, it is not clear which kind of background binders were co-enriched during the aptamer selection for Protein A. The results of additional specificity analyses with the aptamers from group 2 and 6 show that they are able to effectively distinguish Protein A from the functionally related proteins Protein G and Protein L as well (Figure S7). This is in accordance with previously obtained results for PA#2/8. 

Figure 5 also reveals immobilization site effects on the functionality of the Protein A-binding aptamers. As known for PA#2/8, PA-C8 also shows a strongly reduced binding when immobilized via its 5′-end. Both aptamers exhibit similar structural elements and therefore may have a comparable binding behavior. In previous studies, we have comprehensively analyzed the binding behavior of PA#2/8 under different assay designs [30]. We could verify that a free accessible and intact 5′-end of this aptamer is essential for correct folding into the functional structure. The complex three-dimensional conformation is a general prerequisite for the functionality of each aptamer and is important for its specific interaction with the target. Stepwise truncation experiments of PA#2/8 have shown that the stem-loop-structure at the 5′end involving the 5′-primer binding site is essential for binding of the aptamer to Protein A. Removing it leads to a complete loss of function of the aptamer. Related to this, the immobilization of PA#2/8 via the 5′-end also leads to a reduced binding ability. It is known that immobilization may alter the functionality of aptamers in a specific assay. e.g., the molecular flexibility of an aptamer may be restricted impeding its correct folding, or the accessibility of the aptameric binding structure may be limited for the target. Neighborly interactions between aptamers may also interfere with aptamer folding resulting in inhibition of target recognition [36,37,38]. Variation of immobilization density and distance may counter such negative effects. Knowledge about the specific performance of an aptamer at different conditions is crucial for its successful application, e.g., optimal spatial arrangement of the aptamer in the assay or on the sensor surface for most effective target binding and measuring signal formation.

In contrast, the interaction of group 2 aptamers with Protein A is only little affected by the immobilization site.

We could refine the results of our previous SELEX experiment with this medium throughput sequencing approach by expanding the dataset 30-fold and identifying new aptameric sequences in the enriched DNA pool. Several years ago, NGS is increasingly integrated into SELEX experiments in various ways [19]. Schütze et al. performed a SELEX experiment over 10 selection rounds and applied both Sanger sequencing and NGS [20]. They could identify specific binders among several Sanger sequenced clones of the tenth round and were interested in the dynamics of these clones during the whole selection process using high throughput sequencing in all selection rounds. They found, that identified binders start to enrich from round 3, whereas the complexity of the DNA pools drops dramatically after round 4. Unique sequences that occurred after round 5 seem to be derivatives of strongly enriched clones generated by mutation or sequencing artifacts. The authors also found that the frequency of specific binders often reached a maximum until rounds 6 or 7 and then tended to decrease in the following rounds. Interestingly, the most abundant sequences analyzed in the final selection round did not correlate with the strongest binding behavior. Similar observations were described by Berezhnoy et al. They confirmed that the best binders for the given target identified by high throughput sequencing after five SELEX rounds, progressively disappeared in further rounds (in total 16 rounds) while weak binders became more enriched [39].

Other researchers focused on an extremely reduced SELEX process with three down to only one selection round in combination with high throughput sequencing and comprehensive sequence analysis for a rapid aptamer development [40,41]. The application of capillary electrophoresis (CE) provides the possibility to strongly reduce the number of SELEX rounds. CE is known for its high partitioning efficiency and therefore has been successfully introduced in SELEX experiments for the separation of unbound oligonucleotides from target-bound oligonucleotides during the aptamer selection process (as reviewed in [9,15,17,42]). For example, Riley et al. combined CE with NGS [43]. The authors demonstrated the identification of thrombin binding aptamers from a spiked SELEX library during a single round of CE-based selection directly followed by NGS and data analysis for aptamer identification and frequency distribution. The aptamer content could be increased from 0.4% in the original library before selection to >15% in the CE-selected fraction. A similar strategy was applied for the de novo selection of aptamers for vitronectin [44].

More recently, Valenzano et al. applied high throughput sequencing and bioinformatics analysis at specific stages of a multiple round SELEX process where changes of the selection conditions occurred [45]. They were interested in deeply understanding the effects of increasing stringency on the enrichment of target-specific aptamers and their dynamics over the course of 21 SELEX rounds. At the end, they could identify high affinity aptamers for the small molecule tyramine from the largest sequence clusters of the last selection round. Soldevilla et al. combined high throughput sequencing with a strategy of Conserved Motif Accumulation (CMA) [46]. They applied NGS after the last two rounds (6–7) in their SELEX experiment and identified the five most abundant aptamers. After a motif analysis, the authors postulate that aptamer species with a higher accumulation of potential binding motifs are likely to have a higher probability of being better binders.

These different approaches exemplify the multitude of possibilities to integrate next generation sequencing into SELEX experiments with the aim to improve the process of aptamer development. It also underlines that the aptamer development remains a complex process and a simple universal method or strategy does not exist. 

### 2.4. Comparative Affinity Studies of Protein A-Targeting Aptamers

**Figure 6:**
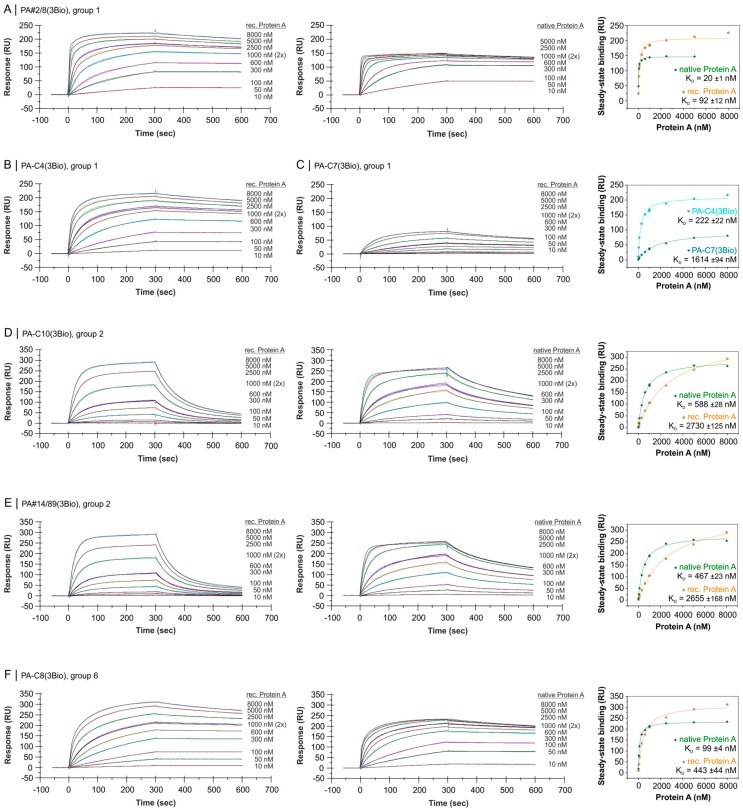
SPR-based interaction analyses with the Biacore X100 instrument regarding the affinity of aptameric sequences from group 1 (**A**–**C**), 2 (**D**,**E**) and 6 (**F**) to Protein A. Biotinylated aptamers were immobilized via the 5′- or 3′-end on the streptavidin-modified sensor surface and a concentration series of recombinant or native Protein A was applied for binding. Black lines represent the fit to bivalent analyte binding model (group 1 and 6 aptamers) or to two state reaction model (group 2 aptamers). The corresponding plots of steady-state binding data from the end of the association phases against analyte concentrations were used to calculate the steady-state affinities (*K_D_*).

Biacore X100 was also used to analyze the affinities of the screened candidate aptamers from group 1, 2 and 6 for their binding to Protein A. Concentration series of recombinant and native Protein A in the range of 10–8000 nM were applied for binding with immobilized aptamers. The best binding aptamer is PA#2/8 (group 1) with steady-state affinities in the low nanomolar range. *K_D_* values of 20 ± 1 nM for native Protein A and 92 ± 12 nM for recombinant Protein A were calculated from saturation curves of binding data at the end of the binding phases (Figure 6A). These *K_D_* values are significantly lower than that described previously and could be achieved by optimizing interaction conditions like thermal equilibration of the aptamers and lowering its immobilization level on the sensor surface. The aptamer is also characterized by a very stable binding to Protein A visualized by a slow dissociation shown in the sensorgrams in Figure 6A.

Besides PA#2/8, two other aptameric sequences, PA-C4 and PA-C7, are very frequent in the group and were therefore analyzed regarding their binding abilities to Protein A. A very similar binding behavior was observed for PA-C4 with a slightly lower affinity of 222 ± 22 nM for recombinant Protein A (Figure 6B). In comparison to the sequence of PA#2/8, the nucleotide exchange in PA-C4 at one position in the sequence between G-rich region 1 and 2 (G_5_AT**A**GAG_5_ → G_5_AT**G**GAG_5_) has only little effect on the binding ability. In contrast, an additional guanine in G-rich region 1 of PA-C7 (G_5_ → G_6_) has a strongly negative effect on the binding to Protein A resulting in a decreased affinity of 1614 ± 94 nM (Figure 6C). This was unexpected, because both size variants of G-rich region 1 are highly frequent among the sequences of group 1 (G_5_ with 141 sequences and G_6_ with 102 sequences). PA-C7 could therefore be the result of a particular type of sequencing error concerning homopolymers as mentioned above.

The relationship between PA#2/8 and PA-C8 (group 6) proved by identified consensus regions and common structural features is further confirmed by comparable association and dissociation behavior during interaction with Protein A (Figure 6F). Slightly higher *K_D_* values of 99 ± 4 nM for native Protein A and 443 ± 44 nM for recombinant Protein A were calculated.

A significant different binding behavior to Protein A was observed for PA-C10 and PA#14/89 (group 2). The binding curves of both 3′-immobilized aptamers are characterized by a fast dissociation of the binding complexes especially after interacting with recombinant Protein A (Figure 6D,E). This indicates an unstable binding complex and results in affinities only in the micromolar range with *K_D_* = 2730 ± 125 nM for PA-C10 and *K_D_* = 2655 ± 168 nM for PA#14/89. The affinities increase strongly if binding to native Protein A is measured, and switch in the nanomolar range with *K_D_* = 588 ± 28 nM and *K_D_* = 467 ± 23 nM, respectively. The 5′-immobilized variants of both aptamers bind Protein A with affinities in a similar range (Figure S8, Table S2).

The group 2 aptamers identified in this work exemplify the need of combined experimental approaches already for functional screening of selected sequences to avoid loss of potent aptamers at an early stage of aptamer developments. In our first study, PA#14/89 was not recognized as a Protein A-binding sequence by a fluorescent assay using target-coated magnetic beads. The SPR-based assay used for functional screening in our current study provides a different assay design and also allows a more differentiated insight into the dynamics of the aptamer-target interactions. The fast dissociation of the formed binding complexes as observed in the SPR-based assays has also affected the bead-based binding assays resulting in a release of most of the aptamers from the binding complexes on the beads during the washing steps before quantifying the bound aptamer fraction. This led to the initial assessment of the selected aptamer as non-binding sequence.

McKeague et al. performed a comprehensive analysis of aptamer binding assays with respect to evaluation of small molecule-targeting aptamers [47]. They stated the need for multiple experimental strategies for aptamer candidate screening and characterization. But there is no universal method or assay applicable to functionally verify each aptamer-target system or to compare them. Moreover, the performance of a specific aptamer may vary under different assay designs, e.g., if the aptamer is used in solution or immobilized, or has to be modified for the specific application. Additionally, each assay has its own inherent sensitivity range, and therefore can limit the aptamer affinity reported as *K_D_* value and reflecting the strength of attraction between aptamer and target. A flexible combination of different assays must be included for screening, characterization, and functional verification of aptamers with balancing the efficiency, parallelization and cost-effectiveness [47]. This is one of the challenging issues in the post-SELEX phase and also matches our observations during several aptamer developments for different classes of targets.

**Figure 7:**
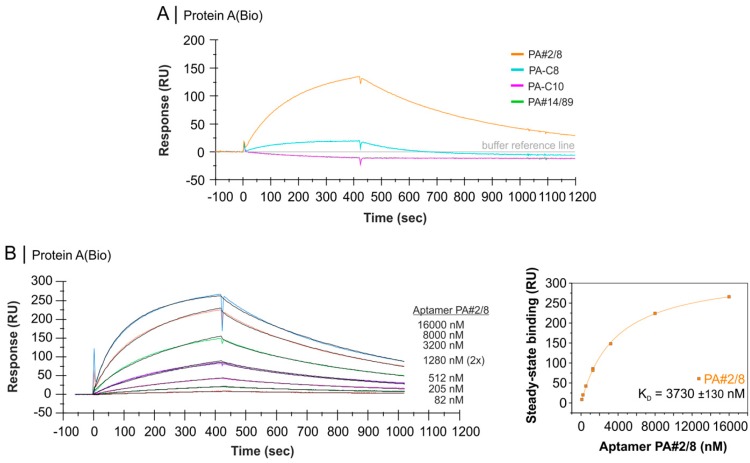
SPR-based interaction analyses with the Biacore X100 instrument applying aptamers as analyte. Biotinylated native Protein A was immobilized as ligand on the streptavidin-modified sensor surface. (**A**) Comparable binding analysis of unmodified candidate aptamers, 2000 nM each; (**B**) A concentration series of aptamer PA#2/8 was applied for binding. Black lines represent the fit to two state reaction model. The corresponding plot of steady-state binding data from the end of the association phases against analyte concentrations were used to calculate the steady-state affinity (*K_D_*).

The binding behaviors of the candidate aptamers were also tested in a reverse experimental setup where the sensor surface was build up by immobilization of biotinylated native Protein A. The unmodified aptamers were applied as analytes. The reverse assembly affects the binding abilities strongly. A good functionality was observed for PA#2/8 as shown in Figure 7A, whereas only weak binding to immobilized Protein A was found for the related aptamer PA-C8. In case of PA#2/8, a concentration series of the aptamer was applied for binding and the dissociation constant was calculated to be in the micromolar range with *K_D_* = 3730 ± 130 nM (Figure 7B). The significant difference in the affinity of the immobilized aptamer and that in solution to Protein A was already discussed previously [30]. Protein A is supposed to be a multivalent target for the aptamer and therefore can cause avidity effects in case of immobilized aptamers. Avidity is much stronger than 1:1 affinity and allows more stable binding complexes.

In contrast, the group 2 aptamers PA-C10 and PA#14/89 are not able to form detectable binding complexes on the sensor surface when applied as analyte in the flow system. These results are in accordance with those shown in Figure 6D,E. As discussed above, the binding complexes of both aptamers with Protein A are relatively unstable hampering the binding of the aptamers to the Protein A-coated sensor surface. Possible stabilizing avidity effects provided by Protein A have no impacts under these assay conditions.

Steric hindrance cannot be excluded and could explain differences in the binding behavior of the aptamers depending on the assay format. The sensor surface was coated with Protein A at a high level, but not fully saturated. Differences between both assay formats also concern Protein A modification. The unmodified protein was used when applied as analyte, but the biotinylated protein was used when applied as ligand immobilized on the sensor surface. Biotin was randomly coupled to Protein A through an aminocaproyl spacer with different extent of labeling (see manufacturer’s description). Biotinylation of Protein A may affect the binding ability of the aptamers by altering original features of the protein or masking the potential binding sites. We will investigate the discrepancy in the binding behavior of the aptamers in our further work.

## 3. Materials and Methods

### 3.1. Materials

Native Protein A from *S. aureus* (P3838), biotinylated native Protein A (P2165), and recombinant Protein A (P7837, expressed in *E. coli*), as well as human serum albumin (HSA, A9511) and bovine serum albumin (BSA, A3059) were purchased from Sigma-Aldrich (Taufkirchen, Germany). Streptavidin (Z704A) was bought from Promega GmbH (Mannheim, Germany). Human Immunoglobulin G (IgG, 009-0102) and rabbit IgG (011-0102-0010) were purchased from Rockland Immunochemicals, Inc. (Limerick, Ireland)/BIOTREND Chemikalien GmbH (Köln, Germany). Recombinant Protein G and Protein L (Pierce 21193 and 21189, expressed in *E. coli*) were purchased from Fisher Scientific (Schwerte, Germany).

5′- or 3′-biotinylated oligonucleotides (aptamers, SELEX library BANK-C) were synthesized by BioSpring GmbH (Frankfurt am Main, Germany). BANK-C represents the starting random oligonucleotide library from which the aptamers were selected for binding to Protein A [30]. Each oligonucleotide in this library is 76 nt long and consists of a variable intern region of 40 nt flanked by specific primer binding regions of 18 nt each at the 5′- and 3′-end: 5′-ATACCAGCTTATTCAATT-N40-ACAATCGTAATCAGTTAG-3′. Unmodified aptamers were purchased from IBA Lifesciences (Göttingen, Germany). Binding buffer was prepared for use in aptamer-target interaction studies (100 mM NaCl, 20 mM Tris-HCl pH 7.6, 10 mM MgCl_2_, 5 mM KCl, 1 mM CaCl_2_).

### 3.2. Next Generation Sequencing (Roche 454 GS FLX System)

A previously selected aptamer pool for bacterial Protein A processed by the classical way of Sanger sequencing [30] was sequenced again by next generation sequencing in this work. The pool was applied as template in a new PCR with the special 454-barcoded Amplicon Forward and Reverse Fusion Primers for library preparation. The primers contain a directional GS FLX Titanium Primer A or Primer B sequence at the 5′-end followed by a unique **DNA Barcode** (10 nt, only Primer A) and the *template specific sequence (18 nt)* at the 3′-end: PrimA-**M06**-*AP10* 5′-CGTATCGCCTCCCTCGCGCCATCAG**ATATCGCGAG***ATACCAGCTTATTCAATT*-3′ and PrimB-*AP30* 5′-CTATGCGCCTTGCCAGCCCGCTCAG*CTAACTGATTACGATTGT-*3′. The PCR was performed using the KOD Hot Start DNA Polymerase Kit from Merck Chemicals GmbH (Darmstadt, Germany) according to the manufacturer’s protocol. The PCR products were applied for preparative agarose gel electrophorese followed by DNA gel extraction with the NucleoSpin^®^ Gel and PCR Clean-up Kit from Macherey-Nagel GmbH & Co. KG (Düren, Germany) according to the user manual. This purified DNA library was than sequenced by Microsynth AG (Balgach, Switzerland) using a Roche 454 GS FLX system as part of a multiplex sample containing eight different libraries separated by unique DNA barcodes. A medium sequencing throughput on 1/16 454 Titanium run was chosen with a typical yield of 20,000–30,000 reads. A total number of 25,494 reads was obtained for the pooled libraries, initially processed and sorted according to the barcodes. This resulted in 2602 reads in a FASTA format file for the aptamer pool selected for Protein A.

### 3.3. Bioinformatics Analysis

The data set of 2602 sequences was pre-processed by identifying the specific 5′- and 3′-primer binding regions in each sequence with a fault tolerance of 25% and removing them using the program *cutadapt* [48]. Subsequently, the sequences were filtered by discarding those longer than 50 nt. As result, a new sequence data set was obtained representing the variable intern regions of the original aptameric sequences. With 77.8%, the majority of these sequences had an expected length of 40 nt and 99% had a length of 40 ± 3 nt.

The pre-processed sequence data set (NGS pool, 2597 sequences) was further analyzed by a two-step clustering and alignment method. Clustering was done using the *cd-hit-454* program [49]. The data set was first clustered at 100% identity (*n* = 10) to make a reduced non-redundant data set. Duplicate sequences and orphans were identified and sorted with respect to the sequence frequency. The non-redundant data set was again clustered at 85% identity (*n* = 6) and finally aligned (CLC Sequence Viewer 7.7 with the alignment tool MUSCEL v3.8.425, QIAGEN Aarhus, Aarhus, Denmark) to identify groups of homologous sequences.

### 3.4. Secondary Structure Prediction

Secondary structure analyses of aptameric sequences were performed by means of the free-energy minimization algorithm according to Zuker [50] using the internet tool mfold at 21 °C with 100 mM [Na^+^] and 10 mM [Mg^2+^] (available at: http://mfold.rna.albany.edu/?q = mfold) [51,52].

### 3.5. SPR-Based Analyses (Biacore X100)

The Biacore X100 instrument together with the Biotin CAPture Kit (GE Healthcare Europe GmbH, Freiburg, Germany) was used for the SPR-based interaction studies between aptamer and target. The Biotin CAPture Kit enables reversible capture of biotinylated ligands and was applied according to manufacturer’s instructions and as previously described [30].

In case of comparative binding analyses, 5′- or 3′-biotinylated aptamers were immobilized on the sensor surface to a nearly saturated level of 1100 ± 100 RU. 2–5 µM of the biotinylated oligonucleotides in running buffer (same as binding buffer + 0.005% surfactant P20) were injected at a flow rate of 5 µL/min and the immobilization levels were adjusted over the injection time. The unselected 5′- or 3′-biotinylated SELEX library BANK-C was immobilized in the reference flow cell by the same way to allow background signal subtraction. Afterwards, 1000 nM Protein A in running buffer was injected at 21 °C at a flow rate of 10 µL/min for 300 s for binding (association phase) followed by a dissociation phase of 300 s. Functionally related proteins like Protein G or Protein L, as well as unspecific proteins like human IgG, rabbit IgG, BSA, HSA or streptavidin were applied for specificity analyses.

In case of affinity measurements, the 5′- or 3′-biotinylated aptamers and library were immobilized to a reduced level of about 400 ± 50 RU by injection of 0.2 µM oligonucleotide solutions in running buffer. In addition, all oligonucleotides were thermally equilibrated (90 °C 8 min followed by 4 °C 10 min and RT) before starting the measuring cycles. Protein A was serially diluted in running buffer to a concentration range of 10–8000 nM and injected for binding as described above. The injection of sample containing 1000 nM Protein A was performed in duplicate at different time points within each experiment.

Double referencing of each data set was achieved by use of the reference sensor surface modified with the SELEX library and injections of running buffer. Data were processed using the BIAevaluation software (GE Healthcare Europe GmbH, Freiburg, Germany). OriginPro 9.0 (OriginLab Corporation, Northampton, MA, USA) was used for plotting the steady-state binding data from the end of the association phases against analyte concentrations. On the basis of these data, saturation curves were obtained and the *K_D_* values (dissociation constants) were calculated by non-linear regression analysis.

For an inverted assay design, biotinylated native Protein A was immobilized as ligand on the sensor surface by injection of 50–100 nM protein in running buffer at a flow rate of 5 µL/min (ligand level 500 ± 20 RU and 700 ± 15 RU, respectively). The reference flow cell was left blank, only streptavidin was captured. 2000 nM of unmodified, thermally equilibrated aptamers were applied as analytes for binding at 21 °C at a flow rate of 10 µL/min for 420 s (association phase) followed by a dissociation phase of 600 s. In case of affinity measurements, aptamers were stepwise diluted in running buffer in a concentration range of 82–16,000 nM and injected for binding. 1280 nM aptamer was injected twice. As described above, the experiments were done under conditions allowing double referencing of the binding data.

## 4. Conclusions

We have shown that the results of our classical SELEX experiment including cloning and Sanger sequencing of the enriched aptamer pool selected for binding to Protein A could be expanded by additionally applying NGS technology. We wanted to compare both data sets and identified two new aptameric sequence groups not present in the Sanger data pool that specifically interact with Protein A. On the other hand, known characteristics of the aptamer pool like high complexity, high portion of orphans, relative frequency distribution of certain sequences, or co-enrichment of background binders were confirmed by the NGS pool. In contrast to the Sanger data pool, the NGS pool is clearly dominated by one sequence group represented by PA#2/8. This aptamer is already known from our previous work and was shown to be the most potent aptamer also in this investigation.

We have chosen a medium throughput NGS approach, which is suitable to replace the labor-intensive combination of cloning and Sanger sequencing to get individual aptamers in classical SELEX experiments. Analyses of the sequence data by basic bioinformatics tools for trimming, clustering and alignment allow ranking and the formation of groups with respect to sequence frequency and similarity. This strategy based on medium throughput NGS is cost-effective, can be implemented easily in various laboratories and provides a good characterization of the enriched sequence pool. But harnessing the whole potential of NGS technologies certainly offers deeper insights into the dynamic process of sequence enrichment during the course of several selection rounds and offers possibilities to optimize the entire aptamer selection process. Instead of end-point evaluation of SELEX experiments, the analysis of the sequence pool after each selection round including monitoring of over-represented sub-sequences, identifying structural motifs or determining round-to-round enrichment of a particular aptamer sequence aims at an early identification of potent aptameric sequences. However, that places higher demands on the high throughput NGS data analysis and requires expert knowledge in the field of bioinformatics or specific software solutions.

The selection of Protein A-binding aptamers was aimed at the provision of affinity molecules for the detection of the bacterial pathogen *S. aureus*. The resulting aptamers differ in certain binding features, but they all interact specifically with Protein A and have a significantly increased affinity to native than to recombinant Protein A. Two of the aptamers (PA#2/8 and PA-C8) are very suitable as capture molecules and in particular PA#2/8 as reporter molecule. We have previously shown that PA#2/8 is functional in different assay designs and is able to recognize intact cells of *S. aureus* [32]. Our next steps will focus on the integration of the aptamers in detection assays, e.g., sandwich-type assays like ELONA or lateral flow assays (LFA), which are suitable for the practicable pathogen detection. In this context, the specific interaction with Protein A without cross-reactivity with immunoglobulins also allows a combined application of these aptamers with antibodies.

## Supplementary Materials

Supplementary materials can be found at http://www.mdpi.com/1422-0067/19/2/642/s1.

## Abbreviations

BSA	Bovine Serum Albumin
CMA	Conserved Motif Accumulation
DNA	Deoxyribonucleic Acid
ELONA	Enzyme-linked Oligonucleotide Assay
HSA	Human Serum Albumin
IgG	Immunoglobulin
*K_D_*	Equilibrium Dissociation Constant
LFA	Lateral Flow Assay
NGS	Next Generation Sequencing
nt	Nucleotide
PCR	Polymerase Chain Reaction
RNA	Ribonucleic Acid
RT	Room Temperature
RU	Resonance Units
SELEX	Systematic Evolution of Ligands by Exponential Enrichment
*S. aureus*	*Staphylococcus aureus*
SPR	Surface Plasmon Resonance Spectroscopy
