# Supplementary material for: Refining the Results of a Classical SELEX Experiment by Expanding the Sequence Data Set of an Aptamer Pool Selected for Protein A
Source: Int J Mol Sci. 2018 Feb 24;19(2):642. doi: 10.3390/ijms19020642 (PMC5855864; doi:10.3390/ijms19020642)
Supplement: Supplementary file 1 [file ijms-19-00642-s001.zip › ijms-265806_revised SuppInfo_Stoltenburg20180209.pdf]

Supplementary Materials

Refining the results of a classical SELEX experiment by expanding the sequence data set of an aptamer pool selected for Protein A

Regina Stoltenburg [1,*], Beate Strehlitz [2]

[1] UFZ – Helmholtz Centre for Environmental Research, Department of Soil Ecology, 06120 Halle, Germany; regina.stoltenburg@ufz.de

[2] UFZ – Helmholtz Centre for Environmental Research, Department Environmental and Biotechnology Centre, 04318 Leipzig, Germany; beate.strehlitz@ufz.de

* Corresponding author

Broadening the sequence data set of the aptamer pool selected for Protein A

Results of sequence analysis of the NGS pool – general survey

**1. clustering** (identity 100%, n = 10)	
no. seq. in NGS pool	2597
no. orphans	1134
no. clustered seq. (clstr size ≥2 seq.)	1463
no. cluster (clstr size ≥2 seq.)	286
no. clstr and orphans	1420

**2. clustering** (identity 85%, n = 6)	
no. seq. in first clustered pool	1420
no. orphans	915
no. clustered seq. (clstr size ≥2 seq.)	505
no. cluster (clstr size ≥2 seq.)	153
no. clstr and orphans	1068

**after two-step clustering**	
no. seq. in NGS pool	2597
no. orphans	789
no. clustered seq. (clstr size ≥2 seq.)	1808

**final grouping by alignment**	
no. seq. in NGS pool	2597
no. ungrouped orphans	782
no. ungrouped clustered seq. (clstr size ≥2 seq.)	1025
no. grouped seq. (group size ≥15 seq.)	790
no. groups (group size ≥15 seq.)	15

Figure S1. Sequence analysis of the NGS pool by a two-step clustering and alignment method. Clustering was done using the *cd-hit-454* program. The data set with 2597 sequences (intern regions of the full-length sequences after pre-processing) was first clustered at 100% identity (n=10) to make a non-redundant data set with 1420 sequences. This non-redundant pool was again clustered at 85% identity (n=6) and finally aligned with the *CLC Sequence Viewer 7.7* program including the alignment tool *MUSCEL v3.8.425* (QIAGEN Aarhus, Denmark). As result of sequence analysis, 15 groups of homologous sequences were identified, which represent all groups with a size of ≥15 sequences, in total 790 sequences from the NGS pool. Smaller groups were not identified. Therefore, 1025 sequences remain ungrouped, but are clustered sequences, and 782 sequences remain orphans.

Results of sequence analysis of the NGS pool – making a non-redundant sequence pool

clstr size	no. clstr	no. seq	cluster C...	aptamers from Sanger data pool identified	new aptamers
1	1134	1134	singleton S... 286 - 1419	PA#4/39 (723), PA#6/46 (767), PA#6/60 (773), PA#14/99 (1028), PA#2/7 (1067), PA#4/40 (1156), PA#10/72 (1167), PA#14/94 (1205)	n.a.
2	134	268	152 - 285	PA#6/47 (153), PA#14/84 (179), PA#10/79 (257)	n.a.
3	43	129	109 - 151	PA#2/14 (118), PA#4/31 (145)	n.a.
4	31	124	78 - 108	PA#10/64 (106)	n.a.
5	15	75	63 - 77		PA-C63-77
6	10	60	53 - 62	PA#6/41 (53), PA#10/66 (58), PA#14/89 (60), PA#10/68 (62)	PA-C54-57, PA-C59, PA-C61
7	12	84	41 - 52	PA#2/13 (43), PA#2/17 (44), PA#14/85 (46)	PA-C41, PA-C42, PA-C45, PA-C47-52
8	10	80	31 - 40	PA#4/28 (40)	PA-C31-39
9	6	54	25 - 30	PA#6/52 (28)	PA-C25-27, PA-C29-30
10	1	10	24		PA-C24
11	2	22	22 - 23	PA#14/93 (22)	PA-C23
12	4	48	18 - 21	PA#10/65 (18), PA#10/78 (20)	PA-C19, PA-C21
13	2	26	16 - 17	PA#6/43 (17)	PA-C16
14	1	14	15		PA-C15
**15**	**1**	**15**	**14**	**PA#2/6**	
**17**	**1**	**17**	**13**		**PA-C13**
**17**	**1**	**17**	**12**		**PA-C12**
**19**	**1**	**19**	**11**		**PA-C11**
**20**	**1**	**20**	**10**		**PA-C10**
**22**	**1**	**22**	**9**		**PA-C9**
**22**	**1**	**22**	**8**		**PA-C8**
**25**	**1**	**25**	**7**		**PA-C7**
**28**	**1**	**28**	**6**	**PA#2/3**	
**33**	**1**	**33**	**5**	**PA#14/82**	
**41**	**1**	**41**	**4**		**PA-C4**
**48**	**1**	**48**	**3**	**PA#4/22**	
**52**	**1**	**52**	**2**	**PA#2/11**	
**54**	**1**	**54**	**1**	**PA#2/8**	
**56**	**1**	**56**	**0**	**PA#4/34**	

Figure S2. Composition of the non-redundant pool (1420 sequences) after the first clustering (100% identity, n=10) of the NGS pool using the *cd-hit-454* program. All identical sequences were clustered (1463 sequences in total) resulting in 286 clusters with at least 2 sequences each, which were ranked with respect to the cluster size. All clusters were numbered according to their rank position (C0-C285). The largest cluster (C0) was found to contain 56 sequences. The remaining 1134 sequences of the NGS pool were unique and therefore called orphans (singleton S286-S1419). Several clusters or unique sequences could be identified as known sequences from the Sanger data pool (all representatives of the 12 sequence groups labelled green and 22 out of 41 orphans). All new sequences from the NGS pool were named according to their cluster number, e.g. PA-C4 comes from cluster C4 with 41 identical sequences.

Group complexities and consensus sequences

Homologous sequences of group 1 of the NGS pool – alignment

seq. name	group 1: intern sequence region (5' → 3')	length (nt)
singleton-S330	-AG-CAAC-ATAGAGGGG-GATAGA-GGGGGT-GGGTTC-TCTCGGACT	42
singleton-S464	-GG-CAAC-ATGAGGGGG--ATAGA-GGGGGTGGGTTAC-TCTCAGCT-	41
singleton-S1268	-AG-CAAC-ATNAGGGGG--ATGA--GGGGGN-GGGTTT-TCTCGGCT-	39
singleton-S1329	-AG-CAAC-ATNAGGGGG--NTAAA--GGGGN-GGGTTC-TCTCGGCT-	39
singleton-S1047	-AG-CAAC-ATGAGGGGG--ATGTA-GGGGGT-GGGTTC-TCTCGGCC-	40
singleton-S451	-AG-CAACAGTGAGGAGG--ATAGA-GGGGGT-GGGTTC-TCTCGGCT-	41
singleton-S302	-AGCTAACGATGACGGGG-GATGGA--GGGGTTGGGTTC-TCTCGGCTA	44
singleton-S359	-CG-CAAC-ATGAGGGGG-GATAGA-GGGGGT-GGGTTC-TTTCGGCT-	41
singleton-S360	-AG-CAAC-ATGAGGGGG--ATAGGGGGGGT-GGGTTC-TCTCGGCT-	41
singleton-S309	-AG-CAAC-ATGAGGGGG-GATAGGAGGGGGGTGGGTTC-TCTCGGCT-	43
singleton-S346	-AG-CAAC-ATGAGGGGG-ATTAGAGGGGGGT-GGGTTC-TCTCGGCT-	42
singleton-S314	-AG-CAAC-ATGAGGGGG-GATAGAGGGGGGT-GGGTTCGTCTCGGCT-	43
singleton-S1352	-AG-CAAC-ATNAGGGGG--ATGA--GGGGGT-GGGTTC-TCTCGACT-	39
singleton-S292	-AG-CAAC-ATNAGGGGGGGATANAGGGGGGTGGGGTTCGTCTCGGCT-	45
cluster-C23	-AG-CAAC-ATNAGGGGG-GATANA-GGGGGT-GGGTTC-TCTCGGCT-	41
cluster-C67	-AG-CAAC-ATNAGGGGG--ATANA-GGGGGT-GGGTTC-TCTCGGCT-	40
singleton-S316	-AG-CAAC-ATNAGGGGGGGATANA-GGGGGT-GGGTTC-TCTCGGCT-	42
cluster-C31	-AG-CAAC-ATNAGGGGG-GATANA-GGGGGTGGGGTTC-TCTCGGCT-	42
singleton-S313	TAG-CAAC-ATNAGGGGG-GATANA-GGGGGTGGGGTTC-TCTCGGCT-	43
singleton-S326	-AG-CAAC-ATNAGGGGG-GATANA-GGGGGTGGGGTTC-TCTCGTCT-	42
singleton-S368	-AG-CAAC-ATNAGGGGG-GATANA-GGGGGT-GGGTTC-TCTCGTCT-	41
singleton-S405	-AG-CAAC-ATNAGGGGG-GATANA-GGGGGT-GGGTTC-TCTCGACT-	41
cluster-C54	-AG-CAAC-ATNAGGGGG-GATAGA-GGGGGT-GGGTTC-TCTCGGCT-	41
singleton-S336	-AG-CAAC-ATNAGGGGG-GATAGAGGGGGGT-GGGTTC-TCTCGGCT-	42
singleton-S1010	-AG-CAAC-ATNAGGGGG-GATAA--GGGGGT-GGGTTC-TCTCAGCT-	40
singleton-S421	-AG-CAAC-ATNAGGGGG-GATAA--GGGGGTGGGGTTC-TCTCGGCT-	41
singleton-S763	-AG-CAAC-ATNAGGGGG-GATAA--GGGGGT-GGGTTC-TCTCGGCT-	40
singleton-S355	-AG-CAAC-ATNAGGGGG-GATGA--GGGGGTGGGGTTC-TCTCGGCC-	41
singleton-S489	-AG-CAAC-ATNAGGGGG--ATGGA-GGGGGT-GGGTTC-TCTCGGCT-	40
cluster-C64	-AG-CAAC-ATNAGGGGG-GATGA--GGGGGTGGGGTTC-TCTCGGCT-	41
cluster-C34	-AG-CAAC-ATNAGGGGG-GATGA--GGGGGT-GGGTTC-TCTCGGCT-	40
singleton-S1387	-AG-CAAC-ATNAGGGGG--ATG---AGGGGT-GGGTTC-TCTCGGCT-	38
singleton-S557	-AG-CAAC-ATGAGGGGG--ATGGA-GGGGGT-GGGTTC-TCCTGGCT-	40
singleton-S423	-AG-CAAC-ATGAGGGGG-GATGGA-GGGAGT-GGGTTC-TCTCGGCT-	41
singleton-S1034	-AG-CAAC-ATGAGGGGG--ATGGA-GGGGGT-GGGTTC-TCTTGGCT-	40
singleton-S334	-AG-CAAC-ATGAGGGGG-GATGGA-GGGGGTGGGGTTC-TCTTGGCT-	42
singleton-S321	-AG-CAACAATGAGGGGG-GATGGA-GGGGGT-GGGTTC-TCTCGGCT-	42
cluster-C152	-AG-CAAC-ATGAGGGGG-GATGGA-GGGGGTGGGGTTC-TCTCGGCT-	42
singleton-S389	-AG-CAAC-ATGAGGGGG--ATGGA-GGGGGTGGGGTTC-TCTCGGCT-	41
• PA-C4 = cluster-C4	-AG-CAAC-ATGAGGGGG--ATGGA-GGGGGT-GGGTTC-TCTCGGCT-	40
cluster-C55	-AG-CAAC-ATGAGGGGG-GATGGA-GGGGGT-GGGTTC-TCTCGGCT-	41
cluster-C272	-AG-CAAC-ATGAGGGGG--ATGGA--GGGGT-GGGTTC-TCTCGGCT-	39
singleton-S1328	-AG-CAAC-ATGAGGGGG--ATGGA--GGGGT-GGGTTC-TCTCGTCT-	39
singleton-S1312	-AG-CAAC-ATGAGGGGG--ATGA--GGGGGT-GGGTCC-TCTCGGCT-	39
singleton-S1060	-AG-CAAC-ATGAGGGGG--ATGGA-GGGGGT-GGGTTC-TCTCGGTT-	40
cluster-C240	-AG-CAAC-ATGAGGGGG--ATGGA-GGGGGT-GGGTTC-TCTCGACT-	40
cluster-C207	-AG-CAAC-ATGAGGGGG--ATGAA-GGGGGT-GGGTTC-TCTCGGCT-	40
singleton-S1064	-AG-CAAC-ATGAGGGGG--ATTGA-GGGGGT-GGGTTC-TCTCGGCT-	40
cluster-C104	-AG-CAAC-ATGAGGGGG--ATAGA-GGGGGT-GGGTTC-TCTCGGTT-	40
singleton-S406	-AG-CAAC-ATGAGGGGG-GATAGA-GGGGGT-GGGTTC-TCTCGGTT-	41
cluster-C139	-AG-CAAC-ATGAGGGGG--ATAGA-GGGGGT-GGGTTC-TCTCGACT-	40
singleton-S442	-AG-CAAC-ATGAGGGGG--ATAGAGGGGGGT-GGGTTC-TCTCGACT-	41
singleton-S455	-AG-CAAC-ATGAGGGGG-GATAGA-GGGGGT-GGGTTC-TCTCGACT-	41
singleton-S412	-AG-CAAC-ATGAGGGGG-GATAGA-GGGGGT-GGGTTC-TCTTGGCT-	41
singleton-S367	-AG-CAAC-ATGAGGGGG-GATAAA-GGGGGT-GGGTTC-TCTCGGCT-	41
singleton-S298	-AG-CAAC-ATGAGGGGGGAGTAGAGGGGGGTGGGGTTC-TCTCGTCT-	44
cluster-C189	-AG-CAAC-ATGAGGGGG--ATAGA-GGGGGT-GGGTTC-TCTCGTCT-	40
singleton-S310	-AG-CAAC-ATGAGGGGG-GATAGAGGGGGGTGGGGTTC-TCTCGGCC-	43
singleton-S301	-AG-CAACGATGAGGGGG-AGTAGA-GGGGGT-GGGTTCCTCTCGGCTT	44
singleton-S339	-AG-CAAC-ATGAGGGGG-GATAGA-GGGGGT-GGGTTCTCTTCGGCT-	42
singleton-S1416	-AG-CAAC-ATGAGGGG---ATAGA--GGGGT--GGTTC-TCTCGGCT-	37
singleton-S1270	-AG-CAAC-ATGAGGGGG--ATAGA-GGGGGT--GGTTC-TCTCGGCT-	39
singleton-S1386	-AG-CAAC-ATGAGGGGG--ATAGA--GGGGT--GGTTC-TCTCGGCT-	38
singleton-S397	-AGACAAC-ATGAGGGGG--ATAGA-GGGGGT-GGGTTC-TCTCGGCT-	41
cluster-C41	-AG-CAAC-ATGAGGGGG-GATAGAGGGGGGTGGGGTTC-TCTCGGCT-	43
**PA#2/8** = cluster-C1	-AG-CAAC-ATGAGGGGG--ATAGA-GGGGGT-GGGTTC-TCTCGGCT-	40
• PA-C7 = cluster-C7	-AG-CAAC-ATGAGGGGG-GATAGA-GGGGGT-GGGTTC-TCTCGGCT-	41
cluster-C154	-AG-CAAC-ATGAGGGGG-GATAGAGGGGGGT-GGGTTC-TCTCGGCT-	42
singleton-S437	-AG-CAAC-ATGAGGGGG--ATAGAGGGGGGT-GGGTTC-TCTCGGCT-	41
cluster-C269	-AG-CAAC-ATGAGGGGG--ATAGA--GGGGT-GGGTTC-TCTCGGCT-	39

G-rich region 1 | G-rich region 2 | G-rich region 3 | G-rich region 4

Figure S3. Alignment of homologous sequences from group 1 the largest group of the NGS pool. Group 1 contains 20 differently sized clusters and 50 orphans (singletons), in total 247 sequences, which are characterized by four G-rich regions. The group is represented by aptamer PA#2/8 (cluster C1 with 54 sequences).

Homologous sequences of group 1 of the NGS pool – size of the identified G-rich regions

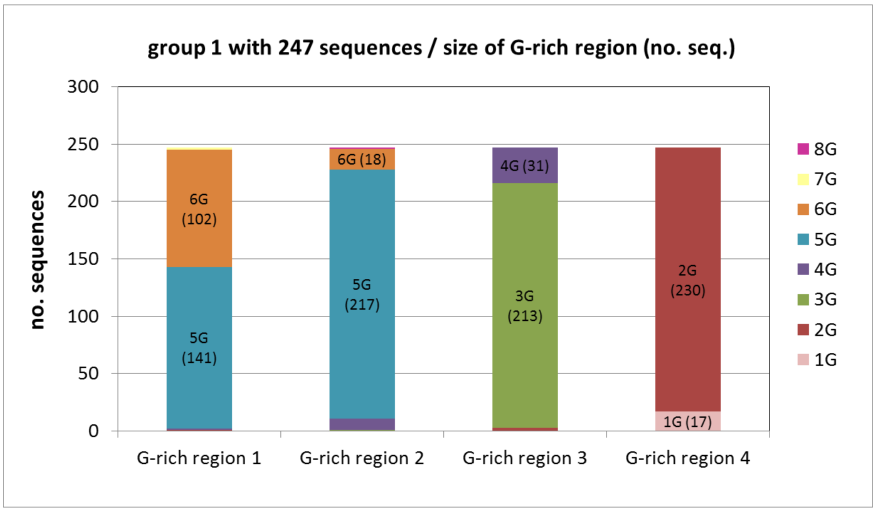

Figure S4. Counting of guanines in each G-rich region of the 247 sequences forming group 1 (with 20 differently sized clusters and 50 orphans). Size of G-stretches (1G-8G) and their frequency are indicated.

Homologous sequences of group 2 and 6 of the NGS pool – alignments

seq. name	group 2: intern sequence region (5' → 3')	length (nt)
	20 / 40	
cluster-C156	-GCGC-ACCACGGGGAGTCGGCCACA-TTTGGAGTTG-TTTTTGC	41
singleton-S449	-GCGC-ACCAC-GGGAGTCGGCCACA-TTTGGAGTTGTTTTTTGC	41
cluster-C21	-GCGC-ACCAC-GGGAGTCGGCCACA-TTTGGAGTTG-TTTTTGC	40
cluster-C280	-GCGC-ACCAC-GGGAGTCGGCCACA-TTTGGAGTTG--TTTTGC	39
**PA#14/89** = cluster-C60	-GGGC-ACCAC-GGGAGTCGGCCACA-TTTGGAGTTG-TTTTTGC	40
cluster-C142	-GTGC-ACCAC-GGGAGTCGGCCACA-TTTGGAGTTG-TTTTTGC	40
cluster-C99	-GGGC-ACCAC-GGGAGTTGGCCACA-TTTGGAGTTG-TTTTTGC	40
cluster-C86	-GTGC-ACCAC-GGGAGTTGGCCACA-TTTGGAGTTG-TTTTTGC	40
singleton-S581	-GCGC-ACCAC-GGGAGTTGGCCACA-TTTGGAGTTG-TTTTTGA	40
singleton-S401	-GCGC-ACCAC-GGGAGTTGGCCACATTTTGGAGTTG-TTTTTGC	41
**PA-C10** = cluster-C10	-GCGC-ACCAC-GGGAGTTGGCCACA-TTTGGAGTTG-TTTTTGC	40
singleton-S1125	-ACGC-ACCAC-GGGAGTTGGCCACA-TTTGGAGTTG-TTTTTGC	40
singleton-S1281	-GCGC-ACCAC-GGGAGTTGGCCACA-TTTGGAGTG--TTTTTGT	39
singleton-S1314	-GTGC-ACCAC-GGGAGTTGGCCACA-TTTGGAGTG--TTTTTGC	39
singleton-S916	-GGGCGACCAC-AGGAGTCGGCCACA-TTTGGAGTG--TTTTTGC	40
cluster-C187	-GGGC-ACCAC-GGGAGTCGGCCACATTTTGGAGTG--TTTTTGC	40
cluster-C77	-GGGC-ACCAC-GGGAGTCGGCCACA-TTTGGAGTG--TTTTTGC	39
singleton-S894	GGGGC-ACCAC-GGGAGTCGGCCACA-TTTGGAGTG--TTTTTGC	40
singleton-S481	-GCGC-ACCAC-GGGAGTTGGCCACA-TTTGGAGTGT-TTTTTGC	40
cluster-C51	-GCGC-ACCAC-GGGAGTTGGCCACA-TTTGGAGTG--TTTTTGC	39
singleton-S333	-GCGC-ACCACGGGGAGTCGGCCACATTTTGGAGTGT-TTTTTGC	42
singleton-S428	-GCGC-ACCACCGGGAGTCGGCCACA-TTTGGAGTGT-TTTTTGC	41
cluster-C52	-GCGC-ACCAC-GGGAGTCGGCCACA-TTTGGAGTG--TTTTTGC	39

seq. name	group 6: intern sequence region (5' → 3')	length (nt)
	20 / 40	
cluster-C282	AGGCCAGATNA-GGGGTGCCCAT-GC-GGG--TGGCTG-CTCC-	37
singleton-S1395	AGGCCAGATNA-GGGGTGCCTAT-GC-GGG--TGGCTG-CTCCA	38
singleton-S534	AGGCCAGATGA-GGGGTGCCTATGGC-GGG--TGGCTGCCTCCA	40
singleton-S1408	AGGCCAGATGA--GGGTGCCTAT-GC-GGG--TGGCTG-CTCCA	37
cluster-C61	AGGCCAGATGA-GGGGTGCCTATGGCGGGG--TGGCTG-CTCCA	40
singleton-S311	AGGCCAGATGAGGGGGTGCCCATGGCGGGGGTGGGCTG-CTCCA	43
cluster-C284	AGGCCAGATGA--GGGTGCCCAT-GC-GGG--TGGCTG-CTCCA	37
cluster-C151	AGGCCAGATGA-GGGGTGCCCAT-GCGGGG--TGGCTG-CTCCA	39
cluster-C108	AGGCCAGATGA-GGGGTGCCCAT-GC-GGG--TGGCTG-CTCCA	38
**PA-C8** =cluster-C8	AGGCCAGATGA-GGGGTGCCCATGGCGGGG--TGGCTG-CTCCA	40
singleton-S1372	AGGCCAGATGA-GGGGTGCC-ATGGCGGGG--TGGCTG-CTCCA	39

Figure S5. Alignment of homologous sequences from group 2 and 6 of the NGS pool. Group 2 contains 12 differently sized clusters and 11 orphans (singletons), in total 85 sequences. This group is represented by aptamer PA-C10 (cluster C10 with 20 sequences) and also contains aptamer PA#14/89 (cluster C60 with 6 sequences). Group 6 contains 6 differently sized clusters and 5 orphans, in total 44 sequences. This group is represented by aptamer PA-C8 (cluster C8 with 22 sequences). Both groups are also characterized by several G-rich regions.

Sequence group 1 and 6 of the NGS pool – consensus regions

seq. name	group 1 and group 6: intern sequence region (5' → 3')	length (nt)
	20 / 40	
PA#2/8	-AGCAACATGAGGGGGATA---GAGGGGGTGGGTTCTCTCGGCT	40
PA-C8	AGGCCAGATGAGGGGTGCCCATGGCGGGGTGGCTGCTCCA----	40
	..GC.A.ATGAGGGG........G..GGGGTGG.T.CTC......	

Figure S6. Alignment of the representatives of sequence group 1 (PA#2/8) and group 6 (PA-C8). Two consensus regions that overlap three G-stretches in both sequences were identified.

Results of sequence analysis of the NGS pool – list of all grouped sequences

See separate PDF-File: "ProtAAptNGS_IJMS-SuppInfoTabS1_201801_Stoltenburg"

Table S1. Groups with ≥15 homologous sequences, which were identified by data analysis of the NGS pool using the two-step clustering and alignment method. Each group consists of a different number of clusters (containing identical sequences) and singletons (unique sequences also called orphans). All grouped sequences are listed.

Functional screening of identified aptamer groups

Specificity

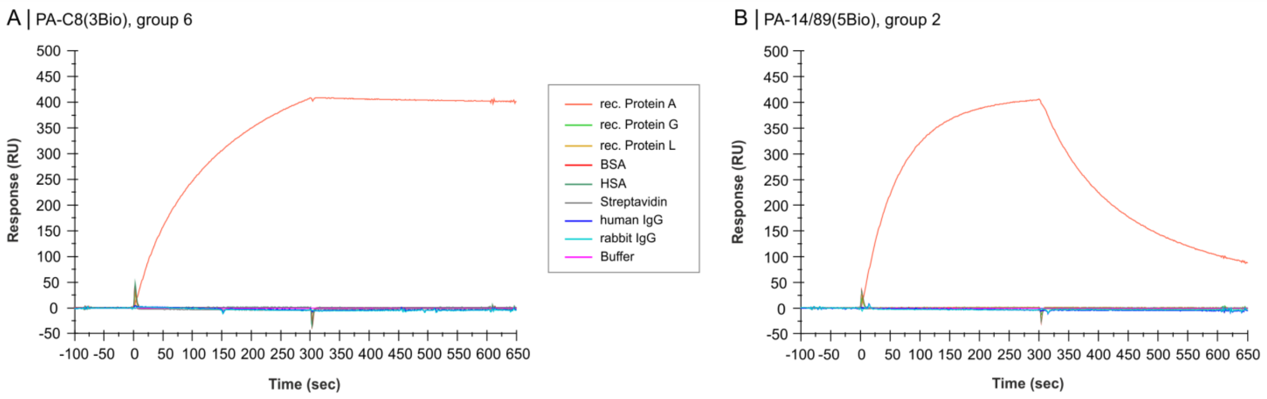

Figure S7. SPR-based interaction analyses with the Biacore X100 instrument regarding the specificity of aptamer PA-C8(3Bio) from group 6 **(A)** and aptamer PA#14/89(5Bio) from group 2 **(B)**. Biotinylated aptamers were immobilized via the 5′- or 3′-end on the streptavidin-modified sensor surface and 1000 nM of different proteins was applied for binding. Double-referenced sensorgrams are shown (reference surface modified with unselected SELEX library, buffer injection).

Comparative affinity studies of Protein A-targeting aptamers

Affinity

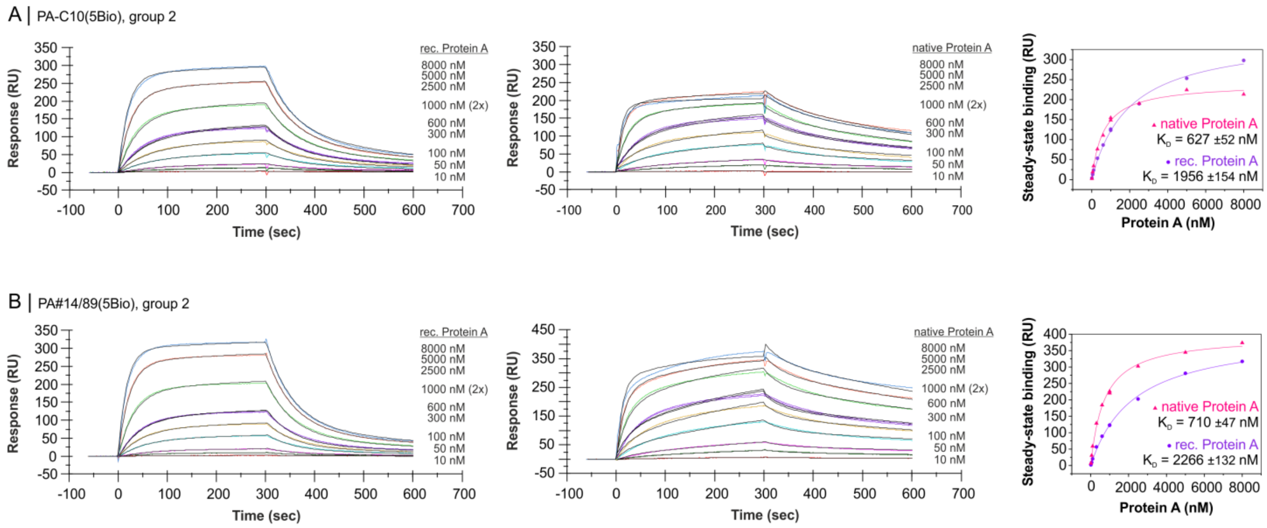

Figure S8. SPR-based interaction analyses with the Biacore X100 instrument regarding the affinity of aptamer PA-C10(5Bio) **(A)** and PA#14/89(5Bio) **(B)** from sequence group 2 to Protein A. Biotinylated aptamers were immobilized via the 5′-end on the streptavidin-modified sensor surface and a concentration series of recombinant or native Protein A was applied for binding. Black lines represent the fit to two state reaction model. The corresponding plots of steady-state binding data from the end of the association phases against analyte concentrations were used to calculate the steady-state affinities (K_D).

Summary of affinity data

Table S2. Overview of steady-state affinities (K_D) of aptameric sequences from group 1, 2 and 6 calculated by SPR-based interaction analyses with the Biacore X100 instrument. Biotinylated aptamers were immobilized via the 5′- or 3′-end on streptavidin-modified sensor surfaces and concentration series of recombinant or native Protein A were applied for binding.

SPR	steady-state affinity / K_D (nM)			
	PA#2/8(3Bio) (represents group 1)	PA-C4(3Bio) (group 1)	PA-C7(3Bio) (group 1)	PA-C8(3Bio) (represents group 6)
**rec. Protein A**	92 ±12	222 ±22	1614 ±94	443 ±44
**native Protein A**	20 ±1	n.a.	n.a.	99 ±4
	PA-C10(5Bio) (represents group 2)	PA-C10(3Bio) (represents group 2)	PA#14/89(5Bio) (group 2)	PA#14/89(3Bio) (group 2)
**rec. Protein A**	1956 ±154	2730 ±125	2266 ±132	2655 ±168
**native Protein A**	627 ±52	588 ±28	710 ±47	467 ±23
