# Supplementary material for: Refining the Results of a Classical SELEX Experiment by Expanding the Sequence Data Set of an Aptamer Pool Selected for Protein A
Source: Int J Mol Sci. 2018 Feb 24;19(2):642. doi: 10.3390/ijms19020642 (PMC5855864; doi:10.3390/ijms19020642)
Supplement: Supplementary file 1 [file ijms-19-00642-s001.zip › ProtAAptNGS_IJMS-SuppInfoTabS1_2018_Stoltenburg.pdf]

Table S1. Groups with ≥15 homologous sequences, which were identified by data analysis of the NGS pool using the two-step clustering and alignment method. Each group consists of a different number of clusters (containing identical sequences) and singletons (unique sequences also called orphans). All grouped sequences are listed.

group (size ≥15 seq.)	non-redundant NGS pool (1. clustering)			aptameric sequence (5' ⟶ 3') ATACCAGCTTATTCAATT ---- intern region ---- ACAATCGTAATCAGTTAG	length of intern region (nt)	aptamer from Sanger pool identified	new aptamer
	cluster or singleton	name	no. seq.				
group 1							
*	**cluster**	**C1**	**54**	**AGCAACATGAGGGGGATAGAGGGGGTGGGTTCTCTCGGCT**	40	**PA#2/8**	
	cluster	C4	41	AGCAACATGAGGGGGATGGAGGGGGTGGGTTCTCTCGGCT	40		PA-C4
	cluster	C7	25	AGCAACATGAGGGGGGATAGAGGGGGTGGGTTCTCTCGGCT	41		PA-C7
	cluster	C23	11	AGCAACATNAGGGGGGATANAGGGGGTGGGTTCTCTCGGCT	41		
	cluster	C31	8	AGCAACATNAGGGGGGATANAGGGGGTGGGGTTCTCTCGGCT	42		
	cluster	C34	8	AGCAACATNAGGGGGGATGAGGGGGTGGGTTCTCTCGGCT	40		
	cluster	C41	7	AGCAACATGAGGGGGGATAGAGGGGGGTGGGGTTCTCTCGGCT	43		
	cluster	C54	6	AGCAACATNAGGGGGGATAGAGGGGGTGGGTTCTCTCGGCT	41		
	cluster	C55	6	AGCAACATGAGGGGGGATGGAGGGGGTGGGTTCTCTCGGCT	41		
	cluster	C64	5	AGCAACATNAGGGGGGATGAGGGGGTGGGGTTCTCTCGGCT	41		
	cluster	C67	5	AGCAACATNAGGGGGATANAGGGGGTGGGTTCTCTCGGCT	40		
	cluster	C104	4	AGCAACATGAGGGGGATAGAGGGGGTGGGTTCTCTCGGTT	40		
	cluster	C139	3	AGCAACATGAGGGGGATAGAGGGGGTGGGTTCTCTCGACT	40		
	cluster	C152	2	AGCAACATGAGGGGGGATGGAGGGGGTGGGGTTCTCTCGGCT	42		
	cluster	C154	2	AGCAACATGAGGGGGGATAGAGGGGGGTGGGTTCTCTCGGCT	42		
	cluster	C189	2	AGCAACATGAGGGGGATAGAGGGGGTGGGTTCTCTCGTCT	40		
	cluster	C207	2	AGCAACATGAGGGGGATGAAGGGGGTGGGTTCTCTCGGCT	40		
	cluster	C240	2	AGCAACATGAGGGGGATGGAGGGGGTGGGTTCTCTCGACT	40		
	cluster	C269	2	AGCAACATGAGGGGGATAGAGGGGTGGGTTCTCTCGGCT	39		
	cluster	C272	2	AGCAACATGAGGGGGATGGAGGGGTGGGTTCTCTCGGCT	39		
	singleton	S292	1	AGCAACATNAGGGGGGGATANAGGGGGGTGGGGTTCGTCTCGGCT	45		
	singleton	S298	1	AGCAACATGAGGGGGGAGTAGAGGGGGGTGGGGTTCTCTCGTCT	44		
	singleton	S301	1	AGCAACGATGAGGGGGAGTAGAGGGGGTGGGTTCCTCTCGGCTT	44		
	singleton	S302	1	AGCTAACGATGACGGGGGATGGAGGGGTTGGGTTCTCTCGGCTA	44		
	singleton	S309	1	AGCAACATGAGGGGGGATAGGAGGGGGGTGGGTTCTCTCGGCT	43		
	singleton	S310	1	AGCAACATGAGGGGGGATAGAGGGGGGTGGGGTTCTCTCGGCC	43		
	singleton	S313	1	TAGCAACATNAGGGGGGATANAGGGGGTGGGGTTCTCTCGGCT	43		
	singleton	S314	1	AGCAACATGAGGGGGGATAGAGGGGGGTGGGTTCGTCTCGGCT	43		
	singleton	S316	1	AGCAACATNAGGGGGGGATANAGGGGGTGGGTTCTCTCGGCT	42		
	singleton	S321	1	AGCAACAATGAGGGGGGATGGAGGGGGTGGGTTCTCTCGGCT	42		
	singleton	S326	1	AGCAACATNAGGGGGGATANAGGGGGTGGGGTTCTCTCGTCT	42		
	singleton	S330	1	AGCAACATAGAGGGGGATAGAGGGGGTGGGTTCTCTCGGACT	42		
	singleton	S334	1	AGCAACATGAGGGGGGATGGAGGGGGTGGGGTTCTCTTGGCT	42		
	singleton	S336	1	AGCAACATNAGGGGGGATAGAGGGGGGTGGGTTCTCTCGGCT	42		
	singleton	S339	1	AGCAACATGAGGGGGGATAGAGGGGGTGGGTTCTCTTCGGCT	42		
	singleton	S346	1	AGCAACATGAGGGGGATTAGAGGGGGGTGGGTTCTCTCGGCT	42		
	singleton	S355	1	AGCAACATNAGGGGGGATGAGGGGGTGGGGTTCTCTCGGCC	41		
	singleton	S359	1	CGCAACATGAGGGGGGATAGAGGGGGTGGGTTCTTTCGGCT	41		
	singleton	S360	1	AGCAACATGAGGGGGATAGGGGGGGGTGGGTTCTCTCGGCT	41		
	singleton	S367	1	AGCAACATGAGGGGGGATAAAGGGGGTGGGTTCTCTCGGCT	41		
	singleton	S368	1	AGCAACATNAGGGGGGATANAGGGGGTGGGTTCTCTCGTCT	41		
	singleton	S389	1	AGCAACATGAGGGGGATGGAGGGGGTGGGGTTCTCTCGGCT	41		
	singleton	S397	1	AGACAACATGAGGGGGATAGAGGGGGTGGGTTCTCTCGGCT	41		
	singleton	S405	1	AGCAACATNAGGGGGGATANAGGGGGTGGGTTCTCTCGACT	41		
	singleton	S406	1	AGCAACATGAGGGGGGATAGAGGGGGTGGGTTCTCTCGGTT	41		
	singleton	S412	1	AGCAACATGAGGGGGGATAGAGGGGGTGGGTTCTCTTGGCT	41		
	singleton	S421	1	AGCAACATNAGGGGGGATAAGGGGGTGGGGTTCTCTCGGCT	41		
	singleton	S423	1	AGCAACATGAGGGGGGATGGAGGGAGTGGGTTCTCTCGGCT	41		
	singleton	S437	1	AGCAACATGAGGGGGATAGAGGGGGGTGGGTTCTCTCGGCT	41		
	singleton	S442	1	AGCAACATGAGGGGGATAGAGGGGGGTGGGTTCTCTCGACT	41		
	singleton	S451	1	AGCAACAGTGAGGAGGATAGAGGGGGTGGGTTCTCTCGGCT	41		
	singleton	S455	1	AGCAACATGAGGGGGGATAGAGGGGGTGGGTTCTCTCGACT	41		
	singleton	S464	1	GGCAACATGAGGGGGATAGAGGGGGTGGGTTACTCTCAGCT	41		
	singleton	S489	1	AGCAACATNAGGGGGATGGAGGGGGTGGGTTCTCTCGGCT	40		
	singleton	S557	1	AGCAACATGAGGGGGATGGAGGGGGTGGGTTCTCCTGGCT	40		
	singleton	S763	1	AGCAACATNAGGGGGGATAAGGGGGTGGGTTCTCTCGGCT	40		
	singleton	S1010	1	AGCAACATNAGGGGGGATAAGGGGGTGGGTTCTCTCAGCT	40		
	singleton	S1034	1	AGCAACATGAGGGGGATGGAGGGGGTGGGTTCTCTTGGCT	40		
	singleton	S1047	1	AGCAACATGAGGGGGATGTAGGGGGTGGGTTCTCTCGGCC	40		
	singleton	S1060	1	AGCAACATGAGGGGGATGGAGGGGGTGGGTTCTCTCGGTT	40		
	singleton	S1064	1	AGCAACATGAGGGGGATTGAGGGGGTGGGTTCTCTCGGCT	40		
	singleton	S1268	1	AGCAACATNAGGGGGATGAGGGGGNGGGTTTTCTCGGCT	39		
	singleton	S1270	1	AGCAACATGAGGGGGATAGAGGGGGTGGTTCTCTCGGCT	39		
	singleton	S1312	1	AGCAACATGAGGGGGATGAGGGGGTGGGTCCTCTCGGCT	39		
	singleton	S1328	1	AGCAACATGAGGGGGATGGAGGGGTGGGTTCTCTCGTCT	39		
	singleton	S1329	1	AGCAACATNAGGGGGNTAAAGGGGNGGGTTCTCTCGGCT	39		
	singleton	S1352	1	AGCAACATNAGGGGGATGAGGGGGTGGGTTCTCTCGACT	39		
	singleton	S1386	1	AGCAACATGAGGGGGATAGAGGGGTGGTTCTCTCGGCT	38		
	singleton	S1387	1	AGCAACATNAGGGGGATGAGGGGTGGGTTCTCTCGGCT	38		
	singleton	S1416	1	AGCAACATGAGGGGATAGAGGGGTGGTTCTCTCGGCT	37		
group 2							
*	**cluster**	**C10**	20	**GCGCACCACGGGAGTTGGCCACATTTGGAGTTGTTTTTGC**	40		**PA-C10**
	cluster	C21	12	GCGCACCACGGGAGTCGGCCACATTTGGAGTTGTTTTTGC	40		
	cluster	C51	7	GCGCACCACGGGAGTTGGCCACATTTGGAGTGTTTTTGC	39		
	cluster	C52	7	GCGCACCACGGGAGTCGGCCACATTTGGAGTGTTTTTGC	39		
	**cluster**	**C60**	6	**GGGCACCACGGGAGTCGGCCACATTTGGAGTTGTTTTTGC**	40	**PA#14/89**	
	cluster	C77	5	GGGCACCACGGGAGTCGGCCACATTTGGAGTGTTTTTGC	39		
	cluster	C86	4	GTGCACCACGGGAGTTGGCCACATTTGGAGTTGTTTTTGC	40		
	cluster	C99	4	GGGCACCACGGGAGTTGGCCACATTTGGAGTTGTTTTTGC	40		
	cluster	C142	3	GTGCACCACGGGAGTCGGCCACATTTGGAGTTGTTTTTGC	40		
	cluster	C156	2	GCGCACCACGGGGAGTCGGCCACATTTGGAGTTGTTTTTGC	41		
	cluster	C187	2	GGGCACCACGGGAGTCGGCCACATTTTGGAGTGTTTTTGC	40		
	cluster	C280	2	GCGCACCACGGGAGTCGGCCACATTTGGAGTTGTTTTGC	39		
	singleton	S333	1	GCGCACCACGGGGAGTCGGCCACATTTTGGAGTGTTTTTTGC	42		
	singleton	S401	1	GCGCACCACGGGAGTTGGCCACATTTTGGAGTTGTTTTTGC	41		
	singleton	S428	1	GCGCACCACCGGGAGTCGGCCACATTTGGAGTGTTTTTTGC	41		
	singleton	S449	1	GCGCACCACGGGAGTCGGCCACATTTGGAGTTGTTTTTTGC	41		
	singleton	S481	1	GCGCACCACGGGAGTTGGCCACATTTGGAGTGTTTTTTGC	40		
	singleton	S581	1	GCGCACCACGGGAGTTGGCCACATTTGGAGTTGTTTTTGA	40		
	singleton	S894	1	GGGGCACCACGGGAGTCGGCCACATTTGGAGTGTTTTTGC	40		
	singleton	S916	1	GGGCGACCACAGGAGTCGGCCACATTTGGAGTGTTTTTGC	40		
	singleton	S1125	1	ACGCACCACGGGAGTTGGCCACATTTGGAGTTGTTTTTGC	40		
	singleton	S1281	1	GCGCACCACGGGAGTTGGCCACATTTGGAGTGTTTTTGT	39		
	singleton	S1314	1	GTGCACCACGGGAGTTGGCCACATTTGGAGTGTTTTTGC	39		

group<br>(size ≥15 seq.)	non-redundant NGS pool (1. clustering)			aptameric sequence (5' → 3')<br>ATACCAGCTTATTCAATT ---- intern region ---- ACAATCGTAATCAGTTAG	length of intern region (nt)	aptamer from Sanger pool identified	new aptamer
	cluster or singleton	name	no. seq.				
**group 3**							
*	**cluster**	**C3**	48	**GCAGTACTGATGAGTGTAGCCGTATGATTATCGTTTGTGG**	40	**PA#4/22**	
	cluster	C12	17	GCAGTACTGATGAGTGTAGCCGTATGATTANCGTTTGTGG	40		
	cluster	C229	2	GCAGTACTGATGAGTGTAGCCGTATGATTATCGTTTGTGC	40		
	cluster	C238	2	GTAGTACTGATGAGTGTAGCCGTATGATTATCGTTTGTGG	40		
	singleton	S327	1	GCGAGTACTGATGAGTGTAGCCGTATGATTACTCGTTTGTGG	42		
	singleton	S383	1	GCAGTACTGATGAGTGTAGCCGTATGATTATCGTTTGTCGG	41		
	singleton	S462	1	GCAGTACTGATGAGTGTAGCCGTATGATTATCGTTTAGTGG	41		
	singleton	S522	1	GCAGTACTGATGAATGTAGCCGTATGATTATCGTTTGTGG	40		
	singleton	S624	1	GGAGTACTGATGAGTGTAGCCGTATGATTATCGTTTGTGG	40		
	singleton	S743	1	GCAGTACTGATGAGTGTAGCCGTATGATTANCGTTTGTGC	40		
	singleton	S1303	1	GCAGTACTGATGAGTGTAGCCGTATGATTANCGTTTNTG	39		
**group 4**							
*	**cluster**	**C0**	56	**CCCCAACGAGTCGATATGTAGCCCACACTCTGATTCGTCC**	40	**PA#4/34**	
	cluster	C57	6	ACCCAACGAGTCGATATGTAGCCCACACTCTGATTCGTCC	40		
	singleton	S297	1	CCCCAACGAGTCGATAATGTAGCCCACACGTCTGTATTCGTCCA	44		
	singleton	S651	1	CCCTAACGAGTCGATATGTAGCCCACACTCTGATTCGTCC	40		
	singleton	S782	1	CCCCAATGAGTCGATATGTAGCCCACACTCTGATTCGTCC	40		
	singleton	S815	1	CCCCAACGAGTCGATATGTAGCTCACACTCTGATTCGTCC	40		
	singleton	S967	1	CCCCAACGAGTCGATATGTAGCCCACACTCTGATTCGTAC	40		
	singleton	S1004	1	GCACAACGAGTCGATATGTAGCCCACACTCTGATTCGTCC	40		
	singleton	S1172	1	CCCCAACGAGTCGATATGTAGCCCACACTCTGATTCGCCC	40		
	singleton	S1304	1	CCCAACGAGTCGATATGTAGCCCACACTCTGATTCGTCC	39		
**group 5**							
*	**cluster**	**C2**	52	**GGAGACGACAAACTATTACGTACTACGGCATGCACTTGGT**	40	**PA#2/11**	
	singleton	S578	1	GGAGACGACAAACTATTACGTACTACGGTATGCACTTGGT	40		
	singleton	S738	1	GGAGACGACAAACTATTACGTACTACGGCATGCATTTGGT	40		
	singleton	S742	1	GCAGACGACAAACTATTACGTACTACGGCATGCACTTGGT	40		
	singleton	S927	1	GGAGACGACAAACTATTACGTACTACGGCATACACTTGGT	40		
	singleton	S1185	1	GTAGACGACAAACTATTACGTACTACGGCATGCACTTGGT	40		
**group 6**							
*	**cluster**	**C8**	22	**AGGCCAGATGAGGGGTGCCCATGGCGGGGTGGCTGCTCCA**	40		**PA-C8**
	cluster	C61	6	AGGCCAGATGAGGGGTGCCTATGGCGGGGTGGCTGCTCCA	40		
	cluster	C108	4	AGGCCAGATGAGGGGTGCCCATGCGGGTGGCTGCTCCA	38		
	cluster	C151	3	AGGCCAGATGAGGGGTGCCCATGCGGGGTGGCTGCTCCA	39		
	cluster	C282	2	AGGCCAGATNAGGGGTGCCCATGCGGGTGGCTGCTCC	37		
	cluster	C284	2	AGGCCAGATGAGGGTGCCCATGCGGGTGGCTGCTCCA	37		
	singleton	S311	1	AGGCCAGATGAGGGGGTGCCCATGGCGGGGGTGGGCTGCTCCA	43		
	singleton	S534	1	AGGCCAGATGAGGGGTGCCTATGGCGGGTGGCTGCCTCCA	40		
	singleton	S1372	1	AGGCCAGATGAGGGGTGCCATGGCGGGGTGGCTGCTCCA	39		
	singleton	S1395	1	AGGCCAGATNAGGGGTGCCTATGCGGGTGGCTGCTCCA	38		
	singleton	S1408	1	AGGCCAGATGAGGGTGCCTATGCGGGTGGCTGCTCCA	37		
**group 7**							
*	**cluster**	**C5**	33	**CCACAACCGAACTCGTAAGACGTATGTAGCCGCCAACTGT**	40	**PA#14/82**	
	cluster	C201	2	CGACAACCGAACTCGTAAGACGTATGTAGCCGCCAACTGT	40		
	singleton	S294	1	CCACAACCGAACTCGTAAGACGTATGTAGCCGCGACTTTGATTCC	45		
	singleton	S408	1	CCACAACCGAACTCGTAAGGACGTATGTAGCCGCCAACTGT	41		
	singleton	S667	1	CCACAACCGAACTCATAAGACGTATGTAGCCGCCAACTGT	40		
	singleton	S1063	1	CCACAACCGAACTCGTAAGACGTATGTAGCCACCAACTGT	40		
**group 8**							
*	**cluster**	**C6**	28	**CGACAAGTGGGCATTACGATTCTAGCCCTGATTATGTTCC**	40	**PA#2/3**	
	cluster	C68	5	CGACAAGTGGGCATTACGATTCTAGCCCTGATTANGTTCC	40		
	singleton	S343	1	CGACAAGTGGGGCATTTACGATTCTAGCCCTGATTATGTTCC	42		
	singleton	S347	1	CGACAAGTGGGCTATTACGATTCTAGCCCTGATTATGTTCC	41		
	singleton	S363	1	CGACAAGTGGGCACTTACGATTCTAGCCCTGATTATGTTCC	41		
	singleton	S375	1	CGACAAGTGGGCATTACGAATTCTAGCCCTGATTATGTTCC	41		
	singleton	S611	1	CGACAAGTGGGCATTATGATTCTAGCCCTGATTATGTTCC	40		
	singleton	S1006	1	CGACAAGTGTGCATTACGATTCTAGCCCTGATTATGTTCC	40		
**group 9**							
*	**cluster**	**C9**	22	**ACCGAGGAGATAACGTTGTAGCCGTCCATCATCTGATTCG**	40		**PA-C9**
	singleton	S446	1	ACCGAGGAGATAACGTTGTAGCCGTCCATCAGTCTGATTCG	41		
	singleton	S751	1	ACCGAGGAGATAACGTTGTAGCTGTCCATCATCTGATTCG	40		
**group 10**							
*	**cluster**	**C14**	15	**ACCGATCACTAGCCGACTAATTGGTTTCCGATCGCAGTCC**	40	**PA#2/6**	
	cluster	C147	3	ACCGATCACTAGCCGACTAATGGTTTCCGATCGCAGTCC	39		
	cluster	C260	2	ACCGATCACTAGCCGACTAATTGGTTTCCGATTGCAGTCC	40		
	singleton	S576	1	ACCGATCACTAGCCGACTAATTGGTTTCCGATCGCAGTCT	40		
	singleton	S1289	1	ACCGATCACTAGCCGACTAATGGTTTCCGATCGCACTCC	39		
**group 11**							
*	**cluster**	**C11**	19	**CGATGGAGCTGATGATTGTTGCCGATCTGACTGTTGTTCC**	40		**PA-C11**
	cluster	C266	2	CGATGGAGCTGATGATTGTTGCCGATCTGACTGTGTTCC	39		
	singleton	S342	1	CGATGGAGCTGATGAGTTGTTGCCGAGTCTGACTGTTGTTCC	42		
**group 12**							
*	**cluster**	**C13**	17	**CCCCTAACGTTACTGGATGTAGTCCGACTAACTTATGCGT**	40		**PA-C13**
	singleton	S574	1	CCCCTAACGTTACTGGATGTAGTTCGACTAACTTATGCGT	40		
	singleton	S779	1	ACCCTAACGTTACTGGATGTAGTCCGACTAACTTATGCGT	40		

group (size ≥15 seq.)	non-redundant NGS pool (1. clustering) cluster or singleton	name	no. seq.	aptameric sequence (5' → 3') ATACCAGCTTATTCAATT ---- intern region ---- ACAATCGTAATCAGTTAG	length of intern region (nt)	aptamer from Sanger pool identified	new aptamer
**group 13**							
*	**cluster**	**C42**	7	**GCAGATTACGCCTTGTAGCCCGCACTGATCTCGATGTTTGG**	41		**PA-C42**
	cluster	C158	2	GCAGATTACGCCTTGTAGCCCGCACTGATCTCGANGTTTGG	41		
	singleton	S320	1	GCAGATTACGCCTTGTAGCCCCGCACTGATCTCGATGTTTGG	42		
	singleton	S370	1	GCAGATTACGTCTTGTAGCCCGCACTGATCTCGATGTTTGC	41		
	singleton	S379	1	GCAGATTACGTCTTGTAGCCCGCACTGATCTCGATGTTTGG	41		
	singleton	S385	1	ACAGATTACGCCTTGTAGCCCGCACTGATCTCGATGTTTGG	41		
	singleton	S394	1	GCAGATTACGCCTTGTAGCCCGCACTGATCTCGANGTTTGC	41		
	singleton	S413	1	GCAGATTACGCCTTGTAGCCCGCACTGATCTCGATGTTTGC	41		
	singleton	S431	1	GCAGATTACGCCTTGTAGCCCGCACTGATCTCGATGTCTGG	41		
**group 14**							
*	**cluster**	**C16**	13	**CCCACGAGTGTAGCCGATTCTTCTGTACTCTTGTCCTCGT**	40		**PA-C16**
	singleton	S291	1	GCAGTACTGATGAGTGTAGCCGATTCTTCTGTACTCTTGTCCTCGT	46		
	singleton	S467	1	CCCACGAGTGTAGCCGATTCTTCGTGTACTCTTGTCCTCGT	41		
**group 15**							
*	**cluster**	**C15**	14	**ACGTGTTGTAGCCGACCCCTGTTGATTGTTTTCCTGTACC**	40		**PA-C15**
	singleton	S1409	1	ACGTGTAGCCGACCCCTGTTGATTGTTTTCCTGTACC	37		

**no. groups 15**		**no. clstr 60**	**no. clstr seq. 677**
		**no. singleton 113**	**no. singleton 113**
			**sum seq. 790**

** = representative clusters of the groups*
cluster = clustered identical sequences after the 1. clustering
singleton = orphans after the 1. clustering
